# Mesenchymal stem cell therapy for doxorubicin cardiomyopathy: hopes and fears

**DOI:** 10.1186/s13287-015-0109-y

**Published:** 2015-06-24

**Authors:** Fernando Ezquer, Jaime Gutiérrez, Marcelo Ezquer, Christian Caglevic, Helio C Salgado, Sebastián D Calligaris

**Affiliations:** Centro de Medicina Regenerativa, Facultad de Medicina, Clínica Alemana-Universidad del Desarrollo, Av. Las Condes 12348, Lo Barnechea, Santiago, 7690000 Chile; Facultad Ciencias de la Salud, Universidad San Sebastián, Lota 2465, 1° piso Edificio A, Providencia, Santiago, 7500000 Chile; Fundación Arturo Lopez Pérez, Rancagua, Providencia, Santiago, 7500000 Chile; Department of Physiology, School of Medicine of Ribeirão Preto, University of São Paulo, Av. Bandeirantes 3900, Monte Alegre, Ribeirão Preto, São Paulo 14049-900 Brazil

## Abstract

Chemotherapy has made an essential contribution to cancer treatment in recent decades despite its adverse effects. As cancer survivors have increased, concern about ex-patient lifespan has become more important too. Doxorubicin is an effective anti-neoplastic drug that produces a cardiotoxic effect. Cancer survivors who received doxorubicin became more vulnerable to cardiac disease than the normal population did. Many efforts have been made to prevent cardiac toxicity in patients with cancer. However, current therapies cannot guarantee permanent cardiac protection. One of their main limitations is that they do not promote myocardium regeneration. In this review, we summarize and discuss the promising use of mesenchymal stem cells for cardio-protection or cardio-regeneration therapies and consider their regenerative potential without leaving aside their controversial effects on tumor progression.

## Introduction

Globally, cancer is the leading cause of death. There were 14.1 million new cases of cancer in 2012, and an increase of up to 22.2 million new cases by 2030 is predicted [1]. On the other hand, the advances in diagnostic methods for early detection of tumors and the associated treatments have increased the cancer survival rate of the global population [2].

Chemotherapy is an essential tool in cancer treatment. However, the use of anti-neoplastic agents has several adverse effects. Doxorubicin, which belongs to the anthracycline family, has been proven to be effective in different tissue-derived cancer diseases, including cancer of the breast, lung, stomach, bladder, and skin. Despite the anti-tumoral properties of doxorubicin, myelosuppression and particularly cardiotoxicity restrict its clinical use [3].

Doxorubicin has been used in oncology treatment since the 1970s. So far, the following risk factors for doxorubicin-induced cardiotoxicity have been reported: female gender, pre-existing cardiac diseases, mediastinal radiation, cumulative anthracycline doses, and co-treatments with 5-fluorouracil, cyclophosphamide, or taxanes [4].

Cardiomyopathy induced by doxorubicin was described at an early stage (that is, during the first 30 days after the start of treatment) with an incidence of 1 % to 2 % and also several years after the end of drug administration [3]. In fact, retrospective clinical studies estimate that within 30 years after cancer treatment, survivors are eight times more likely to die from cardiac causes and 15 times more likely to be diagnosed with congestive heart failure [5]. This long-term cardiotoxic effect is an especially relevant threat to the survivors of childhood cancer. For this reason, the quality of life of cancer survivors becomes an important issue that promotes the investigation of new monitoring strategies for early diagnosis and multi-agent preventive treatments [6].

In this review, we describe the doxorubicin cardiomyopathy at molecular, histological, and functional levels and the strategies to prevent and monitor cardiac damage. Currently, the cardioprotective treatments based on medical guidelines have limitations, which drive researchers to find new ways to solve them. We discuss the potential of mesenchymal stem cell (MSC) therapy to prevent the cardiotoxicity induced by doxorubicin, its incipient and promising results, and the uncertainty about its use in patients with cancer.

## Doxorubicin cardiomyopathy

Pharmacokinetics studies have demonstrated that doxorubicin has a triphasic plasma clearance after intravenous injection, suggesting that doxorubicin uptake is faster than its elimination from the tissues. For this reason, the risk of toxicity depends directly on the steady-state distribution of the drug [7]. Doxorubicin is accumulated mostly in the liver, due to its role in metabolism, followed by the kidney and heart [8]. In addition, pharmacokinetics analysis has shown the distribution of doxorubicin in different tissues in animal models, providing relevant information to better understand the variability of the outcomes in cancer therapy. Studies of tissue distribution of doxorubicin have demonstrated that the dissemination of the drug in cancer tissue is different than in normal tissue for multi-agent factors; for instance, uneven regional vessel distribution in subcutaneous tumors derived from MDA-MB-231 cells, in athymic nude mice, reduced doxorubicin delivery and interaction with cancer cells [8]. Therefore, this vascular factor may produce drug-resistance phenotype in tumors.

Doxorubicin passes through cell membranes by passive diffusion. Inside the cells, doxorubicin accumulates principally in the nucleus and mitochondria (two orders of magnitude) in comparison with the cytoplasmic concentration [9]. The doxorubicin-anti-neoplastic effect is based on its intercalation into DNA and inhibition of a key enzyme (topoisomerase II) for the DNA replication process [10], killing cells under active proliferation, such as cancer cells. The specific mechanisms of doxorubicin cardiotoxicity are complex and remain unclear. However, these mechanisms are related mainly to the excessive production of reactive oxygen species (ROS) in the mitochondria that cause cellular oxidative stress [11]. Mitochondrial ROS production occurs mainly by NADH dehydrogenase oxidation of doxorubicin and chelation with Fe^2+^ [12]. The heart is particularly sensitive to doxorubicin because it has a high density of mitochondria per cardiomyocyte and low capacity for cellular regeneration (compared with other tissues). As a direct and indirect consequence of oxidative stress, doxorubicin impairs Ca^2+^ signaling in mitochondria and sarcoplasmatic reticulum, altering the contraction cycle in cardiomyocytes, producing lipid peroxidation in cell membranes, and inhibiting transcription processes. These effects downregulate the expression of cardiac muscle-specific proteins (for example, myosin light and heavy chains) and mitochondrial proteins (for example, ADP/ATP translocase), leading the cardiomyocyte to a loss of contraction force by mechanical and energetic causes [13].

Huang and colleagues [14], using a pediatric animal model of late-onset doxorubicin-induced cardiotoxicity, concluded that, besides the toxic effects in cardiomyocytes, doxorubicin impaired cardiac progenitor cell (CPC) proliferation and differentiation into cells of cardiac lineages. Moreover, Piegari and colleagues [15] reported that doxorubicin produces a premature senescence in human CPCs (c-kit^+^) and their progeny, reducing regenerative capacity of the heart. On the other hand, De Angelis and colleagues [16] reported that CPC administration improved the cardiac function in an animal model of dilated cardiomyopathy induced by doxorubicin administration. This doxorubicin cytotoxic effect could explain the increased susceptibility of cancer survivors to develop a cardiac disease after many years of anti-cancer drug treatment.

In regard to the mechanism of apoptosis induced by doxorubicin, there is a consensus on the main role of oxidative stress to activate cell death signal pathways. *In vitro* studies using H9c2 myoblast have shown that oxidative stress induced by doxorubicin activates AMPK (a protein kinase considered to be an intracellular sensor of the energy status) that interacts with p53, leading to bax/bad translocation from cytosol to mitochondria and promoting the release of cytochrome *c* and caspases activation [17,18]. On the other hand, it was reported that doxorubicin downregulates the expression of bcl-2, a protein known for its anti-apoptotic properties. The bax/bcl-2 complex has crucial importance in the cell destiny - survival or death - during doxorubicin treatment [19,20]. Oxidative stress is also involved in the activation of the apoptotic pathway p38-MAPK/NF-κB and release of pro-inflammatory cytokines, including interleukin (IL)-1β and IL-6 and tumor necrosis factor-alpha (TNF-α) in H9c2 cells [21].

Doxorubicin also produces oxidative stress in endothelial cells, leading to an increase in endothelial permeability by reduction of nitric oxide production, pro-inflammatory cytokine secretion, and the expression of adhesion molecules [22]. Leukocyte infiltration and neutrophil activation lead to further cytokine secretion, protease release, and oxidative stress production, thereby exacerbating myocardial injury and death [23].

In this way, doxorubicin triggers a cardiac inflammatory response, in which several mechanisms of innate immune response are activated. To find the key molecules involved in doxorubicin-induced inflammation, researchers have used several strategies, including neutralizing antibodies to specific receptors (for instance, Toll-like receptor 4, or TLR4) [24], knockout mice (for example, TLR4 or STAT3) [25,26], or inhibitory agents for the synthesis of pro-inflammatory molecules (for example, prostaglandin E_2_, or PGE_2_) [27]. The results of these experiments have a common conclusion; when the pro-inflammatory response induced by doxorubicin was inhibited, cardiac function was significantly improved, suggesting that the exacerbated response of the immune system accentuated heart damage.

Currently, under a myocardium cell death process in an inflammatory microenvironment, collagen fiber synthesis is promoted, constituting the whole picture of histological markers in doxorubicin cardiotoxicity: loss of muscle fiber, sarcoplasmatic distention, vacuolization of cardiomyocytes, and fibrosis [28].

In adult patients, the structural and functional changes induced by doxorubicin toxicity progress mainly to dilated cardiomyopathy, which is defined as an increase in left ventricle (LV) dimension, thinness of LV walls, and a severe loss of contractility. However, in pediatric patients, a restrictive cardiomyopathy described by normal dimension and wall thickness of LV and an enlargement of auricle and hardening of cardiac muscle generating diastolic dysfunction is more frequent [29].

## Monitoring and treatments to prevent doxorubicin-induced cardiotoxicity

According to current medical guidelines, monitoring of cardiotoxicity for doxorubicin dose (in milligrams per square meter) is performed with echocardiography and multiple gated acquisition scan. A reduction of 10 % in left ventricular ejection fraction (LVEF) from 50 % (basal) is sufficient to suspend the oncologic treatment [30]. However, these non-invasive methods do not detect early heart injury to prevent subsequent cardiac dysfunction or predict patient tolerance to doxorubicin. Therefore, the identification of new biomarkers has been investigated with promising results. Ky and colleagues [31] proposed that early increases of cardiac troponin I and myeloperoxidase biomarkers are useful to estimate the degree of tolerance of each patient to an oncology treatment for breast cancer. Desai and colleagues [32] identified plasmatic microRNAs (miR-34a and miR-150) that correlated with heart injury in a pre-clinical model; this finding may lead to the development of new biomarkers for earlier events in doxorubicin-induced cardiotoxicity before the release of cardiac troponins.

Prevention of cardiotoxicity is managed mainly by monitoring the maximum cumulative dose (that is, 300 to 350 mg/m^2^ for adults and 200 to 250 mg/m^2^ for children [33]) and by using alternative methods for drug delivery, such as pegylated or non-pegylated liposomal doxorubicin, that increase the half-life of the molecule in plasma, reducing cardiac injury. However, patients usually undergo the uncomfortable adverse effects of plantar-palmar erythrodysesthesia and deeper myelosuppression [34]. An alternative to reducing the cardiotoxic effects of doxorubicin is the co-administration of the drug with iron-chelating agents such as dexrazozane; however, its use is restricted to particular cases of adult patients because data from clinical trials in which the administration of this drug enhances the myelosuppressive effects and interferes with the anti-tumor therapy of doxorubicin have been reported [9,35].

Doxorubicin cardiotoxicity is frequently refractory to conventional pharmacologic therapies for cardiac ischemic diseases. Βeta-blockers (for example, metoprolol) and angiotensin-converting enzyme inhibitors (for example, enalapril) are useful to attenuate doxorubicin-induced cardiomyopathy; however, long-term administration should be balanced with their adverse effects such as hypotension, fatigue, and dizziness since their beneficial effects are only transient [36].

## Cell-based therapies for cardiac diseases

Cell-based therapies have a huge potential to treat cardiovascular diseases because of their regenerative properties and safety. Until 2013, approximately 2,000 patients had been enrolled in clinical trials around the world to evaluate different kinds of stem cell therapies showing promising results [37]. In regard to the cell sources, embryonic stem cells (ESCs) are attractive for therapy applications. ESCs can differentiate into cardiomyocytes, which can integrate into the host cardiac tissue and improve the functional performance in animal models of heart damage. However, the ESCs used in pre-clinical trials have strong bioethical restrictions because it is necessary to destroy human embryos for their generation. Additional complications regarding the use of ESCs include the possibility of teratoma formation in the host and the necessary life-long immunosuppressive therapy to prevent graft rejection [38]. In 2006, Takahashi and Yamanaka [39] described a procedure to induce pluripotency in somatic cells, generating a new kind of stem cells with a wide differentiation potential, called induced pluripotent stem cells (iPSCs). Mauritz and colleagues [40] showed that cardiomyocyte administration, obtained from *in vitro* differentiation of iPSCs, improved the cardiac function in an animal model of infarcted heart, suggesting a promising future for iPSC-based therapy. iPSC therapy has the advantage of being free of ethical restrictions; however, owing to their ESC-like properties, they could be tumorigenic [41]. As a result, more investigations are needed to identify new differentiation and purification protocols before they can be used in clinical trials. In 2003, Beltrami and colleagues [42] reported that the adult heart contains CPCs that support the cardiac regeneration process because of their ability to differentiate into cardiomyocyte or endothelial cells. These cells, isolated from cardiac human biopsies, have the capacity to be highly expanded *ex vivo*, allowing their use in cell-based therapy protocols [43]. The regeneration potential of CPCs was demonstrated in animal models of myocardial infarct [44]. At present, CPC-based therapy is being evaluated with favorable results in two clinical trials: SCIPIO (Stem Cell Infusion in Patients with Ischemic Cardiomyopathy) and CADUCEUS (Cardiosphere-Derived Autologous Stem Cells to Reverse Ventricular Dysfunction), the latter in patients with acute myocardial infarct [45–47]. Finally, clinical or pre-clinical studies (or both) testing new treatments for cardiac regeneration have been reported with the use of adult MSCs, including bone marrow MSCs (BMMSCs), adipose tissue-derived MSCs (ASCs), and MSCs from human umbilical cord blood (hUCBs). In this review, we will focus the discussion on MSC therapies.

MSCs have many characteristics that make them a suitable tool for preventive or regenerative myocardium therapies (or both), including prevention of doxorubicin cardiomyopathy. MSCs are self-renewal cells with the potential to differentiate into cells of the adipogenic, osteogenic, and condrogenic lineages. Moreover, in *in vivo* and *in vitro* models, MSCs can express specific cardiomyocyte markers (for example, connexin 43 and N-cadherin) [48,49]. However, when MSCs were administered by either local or systemic routes, their myocardial homing capacity was weak. For this reason, it is accepted that differentiation into cardiomyocyte is not a relevant mechanism in myocardium regeneration [50]. On the other hand, it was reported that MSCs are attracted to the damaged organs by a chemotaxis process in which MSCs recognize molecules overexpressed in damaged tissues - for example, stromal cell-derived factor-1 and monocyte chemoattractant protein-1 - by interaction with the C-X-C chemokine receptor type 4 and 1 and chemokine receptor type 2 surface receptors [50], leading to a selective homing after systemic administration.

MSCs secrete paracrine factors such as insulin-like growth factor, hepatocyte growth factor, endothelin-1, and basic fibroblast growth factor (with proliferative and anti-apoptotic properties), vascular endothelial growth factor and platelet-derived growth factor (with angiogenic properties), and matrix metallopeptidase-9 (with anti-fibrotic properties) [51,52]; all are involved in the regenerative and cardiac remodeling process. Indeed, MSCs stimulate host CPC proliferation and differentiation and enhance cardiomyocyte cell cycling, mechanisms that could attenuate the long-term cardiotoxic effect of doxorubicin [53,54].

MSCs have been defined as hypoimmunogenic cells because they are not rejected by the recipient’s immune system, even if they come from a non-histocompatible individual [55], allowing allogeneic transplantation therapies.

MSCs also have anti-inflammatory properties through the activation, suppression, migration, or differentiation of specific immune system cells, including T cells, natural killer cells, B cells, macrophages, dendritic cells, and neutrophils, by the secretion of several immune regulators, including transforming growth factor-beta, IL-4, IL-6, IL-10, PGE_2_, and indoleamine 2,3-dioxygenase [56]. The role played by MSCs inside the myocardium during the inflammatory process (induced by infection, metabolic disorders, or chemotherapies) is very difficult to elucidate; however, it is known that Toll-like receptors (for example, TLR3 and TLR4) expressed in MSCs have a key role in the modulation of the inflammatory process [57].

In regard to oxidative stress, the main cause of doxorubicin-induced cardiotoxicity, it was reported that MSCs could manage elevated tissue oxidative stress by reducing ROS-induced apoptosis and modifying the redox microenvironment [58]. Finally, given their technical aspects, MSCs have the advantage that their isolation and *ex vivo* expansion are quite simple and secure from external contamination [59].

## Mesenchymal stem cell therapy for doxorubicin cardiomyopathy

In regard to the development of cell-based therapies to prevent doxorubicin cardiotoxicity or to induce the regeneration of the damaged heart, the investigation is still at pre-clinical stages. Under a regenerative therapy hypothesis, MSCs are administered after an established dilated cardiomyopathy, whereas under a preventive therapy hypothesis, MSCs are transplanted before or during doxorubicin treatment (Table 1). It was reported that the local administration of BMMSCs after 4 weeks of doxorubicin treatment did not improve cardiac function [60]; however, when the BMMSC administration was performed 2 weeks after doxorubicin administration, it generated a significant improvement in LVEF [61]. In a rat model of dilated cardiomyopathy, the intravenous administration of BMMSCs 2 weeks after doxorubicin treatment only reduced myocardium fibrosis [62], but when 10 doses of BMMSCs (one per day) were given intravenously 10 weeks after the doxorubicin treatment, cardiac contractility was improved whereas myocardium fibrosis and LV diameter were reduced. These effects were associated with cardiac remodeling by the downregulation of the renin-angiotensin-aldosterone system [63]. The systemic administration of hUCBs after 2 weeks of doxorubicin treatment also reduced heart weight and cardiac fibrosis but without reported functional data [49]. Di and colleagues [64] reported that hUCBs significantly prevent cardiac dysfunction when they were administered intravenously during chemotherapy. Finally, when ASCs were administered before the doxorubicin chemotherapy, Oliveira and colleagues [65] reported a partial cardioprotective effect. According to the literature, the use of this kind of cell-based therapy is highly versatile because almost all therapies were successful (partial recovery or maintenance of cardiac function) given diverse factors, including (i) time and route of administration of MSCs, (ii) number of doses of MSCs, (iii) source of MSCs, and (iv) grade of cardiac injury induced by doxorubicin. Unfortunately, the duration of the beneficial effect induced by MSC administration has not been tested.

**Table 1:** Cell-based therapies with mesenchymal stem cells for doxorubicin cardiomyopathy

Cell-based therapy hypothesis	Cell type/type of transplantation	Number of cells administered	Delivery route/ time of administration	Animal model	Doxorubicin treatment/ route of administration	Method of cardiac diagnosis	Increase in LVEF versus control (percentage)	References
Regeneration	BMMSC/autologous	1 × 10^7^	Intracoronary/4 weeks after Dox treatment	Rabbit	2 mg/kg per week for 8 weeks/intraperitoneal	Echocardiography	3 (not significant)	[60]
Regeneration	BMMSC/autologous	1.5-2.0 × 10^6^	Epimyocardial/2 weeks after Dox treatment	Rabbit	3 mg/kg for 6 weeks/intraperitoneal	Echocardiography	9 (*P* < 0.002)	[61]
Regeneration	BMMSC/heterologous	5 × 10^6^	Intravenously/2 weeks after Dox treatment	Rat	Three doses of 2.5 mg/kg per week for 2 weeks/intraperitoneal	ND		[62]
Regeneration	BMMSC/ heterologous	5 × 10^6^	Intravenously (one injection per day, 10 times)/ 10 weeks after Dox treatment	Rat	2.5 mg/kg per week for 6 weeks/intraperitoneal	Echocardiography	13 (*P* < 0.05)	[63]
Regeneration	hUCB/xenograft	2.5 × 10^6^	Intravenously/2 week after Dox treatment	Mice	400 ng/kg per minute/oral	ND		[49]
Prevention	hUCB/xenograft	1 × 10^6^	Intravenously/at the end of each Dox cycle	Mice	Three cycles of three doses of 2 mg/kg per week/intraperitoneal	Echocardiography	10 (*P* < 0.05)	[64]
Prevention	ASC/heterologous	3 × 10^6^	Intravenously/3 days before Dox treatment	Rat	5 mg/kg per week for 4 weeks/intraperitoneal	Echocardiography	13 (not significant)	[65]

ASC, adipose tissue-derived mesenchymal stem cell; BMMSC, bone marrow mesenchymal stem cell; Dox, doxorubicin; hUCB, mesenchymal stem cell from human umbilical cord blood; LVEF, left ventricular ejection fraction; ND, not determined

Doxorubicin also has a toxic effect in endogenous MSCs. Oliveira and colleagues [66] reported that BMMSCs, isolated from rats that received doxorubicin, have a lower proliferation rate and lower differentiation capacity (in comparison with cardiomyocytes), suggesting that autologous MSC transplantation to treat doxorubicin cardiomyopathy is not a suitable option for patients after doxorubicin treatment. Moreover, intravenous administration of allogeneic BMMSCs should be performed when plasmatic doxorubicin concentration is under 1 μM in order to reduce a direct cytotoxic effect [67].

**Fig. 1:**
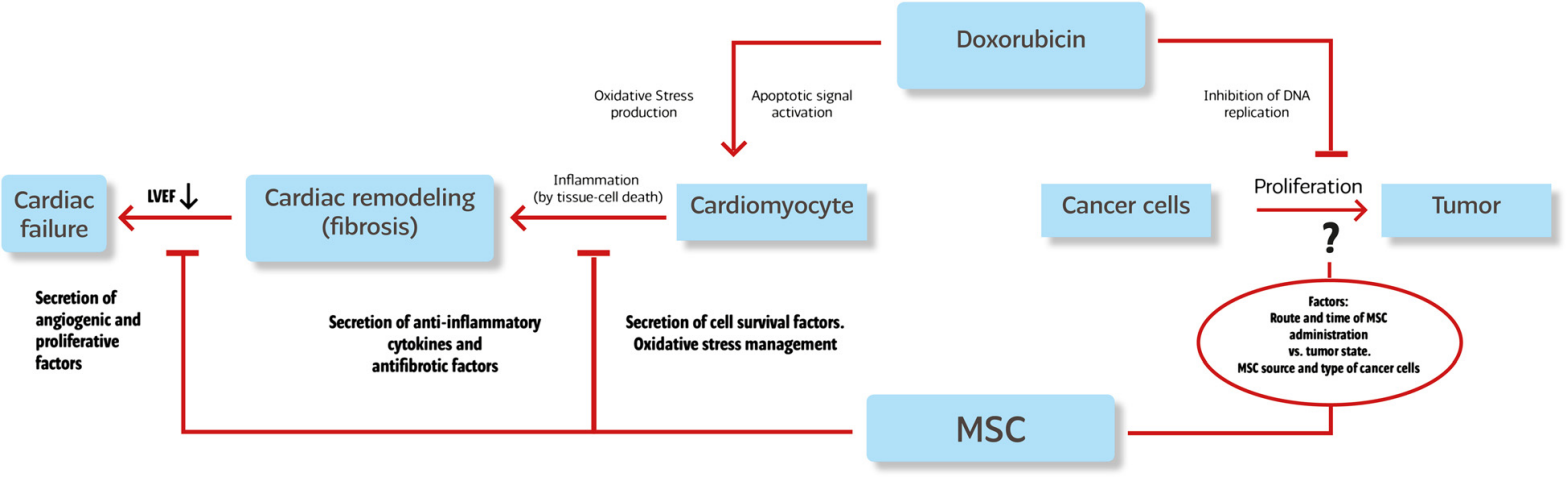
Schematic representation of therapeutic targets of mesenchymal stem cells (MSCs) in doxorubicin cardiomyopathy and tumor progression. LVEF, left ventricular ejection fraction

The systemic administration of MSCs could have integral beneficial effects in patients with cancer. Zoja and colleagues [68] demonstrated that BMMSCs could preserve podocyte viability, reducing glomerular inflammation and sclerosis in an animal model of doxorubicin-induced nephropathy. Additionally, the inflammatory suppressive activity of MSCs could balance the inflammation induced by doxorubicin (i) in the brain, reducing TNF-α production by microglial cells [69], and (ii) in the liver, managing tissue-derived oxidative stress [70] (Fig. 1).

In regard to the use of MSC therapy to prevent or revert the cardiotoxicity effect of anti-cancer drugs such as daunomycin, idarubicin, mitoxantrone (anthracyclines), 5-fluorouracil (anti-metabolite), or cyclophosphamide (alkylating agent), we also expect a beneficial effect in cardiac function because these drugs have a common mechanism of toxicity in cardiomyocytes (excessive ROS production by mitochondria, leading to apoptosis), which is also described in doxorubicin toxicological studies [71–73].

## Mesenchymal stem cell and cancer

It has been postulated that the regenerative potential of MSCs may be a negative feature in patients with cancer. In fact, there is a controversial point of view about the role of MSCs in cancer. Pre-clinical studies reported that MSCs could promote or inhibit tumor growth [74]. Many mechanisms have been associated with these opposite effects, such as vascular support, apoptosis modulation, chemokine signaling, and immune system modulation [36]. In experimental models of cancer in which doxorubicin is also present, the results about the role of MSCs are also contradictory. Human ASC-derived conditioned medium promoted the resistance of MDA-MB-231 cells to doxorubicin [75]; however, human ASCs inhibited the proliferation of MCF-7 cells *in vitro* [76] and increased the sensitivity of cells from a mammary tumor (SKBR3) to doxorubicin [77]. BMMSC-derived conditioned medium improved the viability of 4 T1 cells (mammary adenocarcinoma murine cells) in the presence of doxorubicin; likewise, when BMMSCs were co-injected with 4 T1 cells in the mammary fat pad of mice, BMMSCs inhibited drug-induced apoptosis of tumor cells [78]. However, when hUCBs were injected intravenously in a murine model of pre-established human colon carcinoma treated with doxorubicin, they did not alter drug-anti-tumoral efficiency [64]. On the other hand, MSCs have a positive chemotaxis for tumor cells but this property is independent of tumor growth capacity. Taking advantage of this propriety, many studies have proposed MSCs as a vehicle for delivery of anti-cancer drugs [79]. In summary, it seems that the final result (carcinogenic or anti-tumoral role of MSCs) depends on the microenvironment generated by the specific interaction between cancer cells and MSCs during tumor development (Fig. 1). Further investigation is needed to elucidate the molecular mechanisms of communication between MSCs with cancer cells and with immune system cells.

When human MSCs are properly *ex vivo*-expanded (that is, not forced to cell stress and non-exhausted), no tumoral transformation has been reported. Indeed, no association between autologous or allogeneic MSC administration and tumor formation was found in 36 clinical studies (phase I and II) reported by the Canadian Critical Care Trials Group [80]. However, Kudo-Saito [81], in a study of mice and humans with cancer metastasis, recently identified an MSC subpopulation that aggravates tumor progression, suggesting that MSCs could have a spontaneous tumorigenic potential *in vivo*. Thus, a longer follow-up is required to draw a final conclusion.

## Conclusions

The quality of life of cancer survivors is an emergent topic in the scientific community for the consequences of the adverse events induced by chemotherapy. Therefore, doxorubicin-induced cardiotoxicity is still a relevant issue for oncologic treatment, particularly in pediatric patients. The use of cardiovascular disease therapies based on MSCs is safe, and the myocardium regeneration achieved has a promising impact on the recovery of cardiac function. In animal models, MSC administration could prevent myocardium injury induced by doxorubicin and regenerate the damaged tissue. In addition, owing to the pleiotropic effects of MSCs, their administration could have beneficial effects on extra-cardiac organs. However, in cancer applications, the use of MSCs is still controversial. More pre-clinical studies are needed to better predict the final outcome of the reciprocal influence of cancer cells and MSCs, which is dependent on the source and route of MSC administration and the state and grade of tumor growth. Additionally, clinical trials using MSC therapy may be considered in patients after surgical tumor removal, in order to prevent a heart vulnerability to cardiac diseases in cancer survivors.

## Abbreviations

ASC: adipose tissue-derived mesenchymal stem cell
BMMSC: bone marrow mesenchymal stem cell
CPC: cardiac progenitor cell
ESC: embryonic stem cell
hUCB: mesenchymal stem cell derived from human umbilical cord blood
IL: interleukin
iPSC: induced pluripotent stem cell
LV: left ventricle
LVEF: left ventriclar ejection fraction
MSC: mesenchymal stem cell
PGE_2_: prostaglandin E_2_
ROS: reactive oxygen species
TLR4: Toll-like receptor 4
TNF-α: tumor necrosis factor-alpha
